# Characterization of a novel type of carbonic anhydrase that acts without metal cofactors

**DOI:** 10.1186/s12915-021-01039-8

**Published:** 2021-05-18

**Authors:** Yoshihisa Hirakawa, Miki Senda, Kodai Fukuda, Hong Yang Yu, Masaki Ishida, Masafumi Taira, Kazushi Kinbara, Toshiya Senda

**Affiliations:** 1 Faculty of Life and Environmental Sciences, University of Tsukuba, 1-1-1 Tennodai, Tsukuba, Ibaraki 305-8572 Japan; 2 Structural Biology Research Center, Institute of Materials Structure Science, High Energy Accelerator Research Organization (KEK), 1-1 Oho, Tsukuba, Ibaraki 305-0801 Japan; 3 Graduate School of Life and Environmental Sciences, University of Tsukuba, 1-1-1 Tennodai, Tsukuba, Ibaraki 305-8572 Japan; 4 School of High Energy Accelerator Science, SOKENDAI, 1-1 Oho, Tsukuba, Ibaraki 305-0801 Japan; 5 Applied Research Laboratory, Radiation Science Center, High Energy Accelerator Research Organization (KEK), 1-1 Oho, Tsukuba, Ibaraki 305-0801 Japan; 6 Support Center for Accelerator Science and Technology, High Energy Accelerator Research Organization (KEK), 1-1 Oho, Tsukuba, Ibaraki 305-0801 Japan; 7 School of Life Science and Technology, Tokyo Institute of Technology, 4259 Nagatsuta-cho, Midori-ku, Yokohama, 226-8501 Japan; 8 Faculty of Pure and Applied Sciences, University of Tsukuba, 1-1-1 Tennodai, Tsukuba, Ibaraki 305-8572 Japan

**Keywords:** Carbonic anhydrase, Chlorarachniophytes, Convergent evolution, Crystal structure, Cyanobacteria, Metal cofactor, Microalgae, Plastid

## Abstract

**Background:**

Carbonic anhydrases (CAs) are universal metalloenzymes that catalyze the reversible conversion of carbon dioxide (CO_2_) and bicarbonate (HCO_3_^-^). They are involved in various biological processes, including pH control, respiration, and photosynthesis. To date, eight evolutionarily unrelated classes of CA families (α, β, γ, δ, ζ, η, θ, and ι) have been identified. All are characterized by an active site accommodating the binding of a metal cofactor, which is assumed to play a central role in catalysis. This feature is thought to be the result of convergent evolution.

**Results:**

Here, we report that a previously uncharacterized protein group, named “COG4337,” constitutes metal-independent CAs from the newly discovered ι-class. Genes coding for COG4337 proteins are found in various bacteria and photosynthetic eukaryotic algae. Biochemical assays demonstrated that recombinant COG4337 proteins from a cyanobacterium (*Anabaena* sp. PCC7120) and a chlorarachniophyte alga (*Bigelowiella natans*) accelerated CO_2_ hydration. Unexpectedly, these proteins exhibited their activity under metal-free conditions. Based on X-ray crystallography and point mutation analysis, we identified a metal-free active site within the cone-shaped α+β barrel structure. Furthermore, subcellular localization experiments revealed that COG4337 proteins are targeted into plastids and mitochondria of *B. natans*, implicating their involvement in CO_2_ metabolism in these organelles.

**Conclusions:**

COG4337 proteins shared a short sequence motif and overall structure with ι-class CAs, whereas they were characterized by metal independence, unlike any known CAs. Therefore, COG4337 proteins could be treated as a variant type of ι-class CAs. Our findings suggested that this novel type of ι-CAs can function even in metal-poor environments (e.g., the open ocean) without competition with other metalloproteins for trace metals. Considering the widespread prevalence of ι-CAs across microalgae, this class of CAs may play a role in the global carbon cycle.

**Supplementary Information:**

The online version contains supplementary material available at 10.1186/s12915-021-01039-8.

## Background

Carbonic anhydrase (CA, EC 4.2.1.1) is a well-studied enzyme that catalyzes the interconversion between carbon dioxide (CO_2_) and bicarbonate (HCO_3_^-^) [1, 2]. CAs are universally present in all three domains of life, and eight classes of CAs (α, β, γ, δ, ζ, η, θ, and ι) have been identified so far [3, 4]. θ-CAs structurally resemble β-CAs in the overall architecture [5, 6]. These classes are thought to have evolved convergently, as there is no significant homology in their primary sequences. Although α-, β-, and γ-CAs are widespread in diverse lineages of eukaryotes and prokaryotes, δ-, ζ-, and η-CAs are found in limited species of microalgae and parasitic protists [3, 7]. All known CAs contain a metal cofactor (mostly Zn^2+^, rarely Cd^2+^, Co^2+^, Fe^2+^, and Mn^2+^) [1, 4, 8, 9], which is assumed to play a central role in catalysis; a metal-bound hydroxide ion is considered to act as a nucleophile for attacking CO_2_ to generate HCO_3_^-^ [2]. As a result of convergent evolution, distinct CA classes share structural traits in their active sites; a metal ion is coordinated by three histidine ligands in α-, γ-, and δ-CAs [10, 11], by two histidine residues and one glutamine in η-CA [12], and by one histidine and two cysteines in β-, ζ-, and θ-CAs [5, 6, 13, 14]. CAs are localized in various subcellular compartments (e.g., cytoplasm, periplasm, flagella, mitochondria, and plastids) and play key roles in multiple biological processes associated with pH control, respiration, photosynthesis, and carbon metabolism [15–18]. Therefore, CAs constitute essential enzymes for carbon-based life forms.

In the present study, we report a novel type of CAs that has been characterized in the eukaryotic microalga *Bigelowiella natans* and the cyanobacterium *Anabaena* sp. PCC7120. A previously uncharacterized protein “COG4337,” classified in clusters of orthologous groups (COGs), has been found in various microorganisms [19]. Our biochemical assays demonstrated recombinant COG4337 proteins to be able to catalyze CO_2_ hydration. Surprisingly, they showed the activity under metal-depleted conditions, unlike other known CAs. We also proposed a possible catalytic model for CO_2_ hydration based on their X-ray structures and point mutation analysis. COG4337 proteins are the first example of CAs, to our knowledge, that can function under limiting environments of trace metals.

## Results and discussion

### Frequent occurrence of COG4337 proteins

COG4337 proteins have been found in various prokaryotes and eukaryotic microalgae including ecological important species [19]. NCBI BLAST searches (June 13, 2020) detected this uncharacterized protein in thousands of prokaryotic genomes from proteobacteria (2954 hits), cyanobacteria (98 hits), firmicutes (375 hits), bacteroidetes (89 hits), and several Archaea. Additionally, phylogenetically diverse eukaryotic algae (e.g., dinoflagellates, haptophytes, ochrophytes, prasinophytes, rhodophytes, euglenophytes, and chlorarachniophytes) were found to possess COG4337 homologs. COG4337 proteins are characterized by a conserved domain composed of approximately 160 amino acids. Interestingly, prokaryotic genes encode only a single domain whereas eukaryotic sequences often carry multiple repeat domains (up to five) (Additional file 1: Table S1). To understand the evolution of COG4337 proteins, we constructed phylogenetic trees using conserved domain sequences (Additional file 2: Figure S1). Owing to the short alignment, detailed phylogenetic relationships were poorly resolved. Eukaryotic and prokaryotic sequences were divided into two clades, and some eukaryotic sequences were found to be patchily distributed within the prokaryotic clade, probably due to multiple independent gene transfers from bacteria to eukaryotes. Domain sequences generally have moderate variations among repeats in most eukaryotes, and the tree suggests that domain duplication events have occurred several times before diversification of species in each algal lineage.

### Enzyme activity

**Fig. 1:**
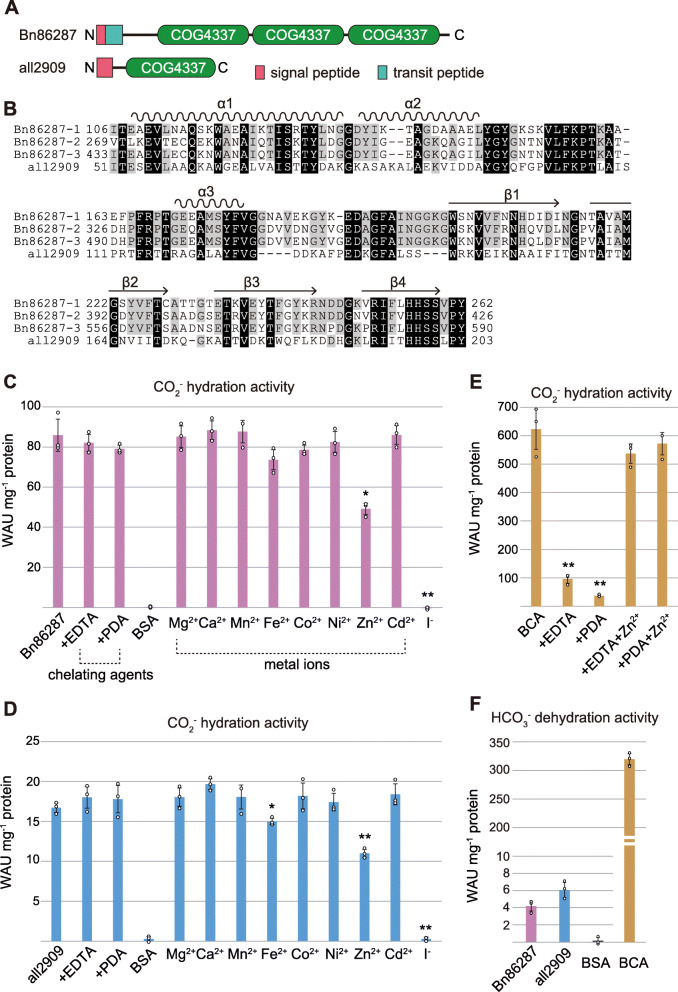
CA activity of COG4337 proteins. **a** Schematic images of Bn86287 and all2909 proteins. **b** Sequence alignment of COG4337 domains extracted from Bn86287 and all2909. Amino acids conserved in all and three of the four domains are shaded in black and gray, respectively. Positions of α-helices and β-strands estimated by X-ray crystallography are shown above the alignment. **c**–**e** CO_2_ hydration activity of Bn86287, all2909, and α-class bovine CA (BCA) under various conditions: +EDTA, proteins were treated with 50 mM EDTA and 6 M urea; +PDA, proteins were treated with 50 mM 2,6-pyridinedicarboxylic acid (PDA); chemical symbols, metal ions were added to protein solution; I^−^, 1 mM KI was added to the reaction solution as an inhibitor. Bovine serum albumin (BSA) was used as a negative control. Significant differences compared to non-treated samples were determined by the two-tailed Student’s *t* test (*P < 0.02, **P < 0.01). In the graphs, error bars represent the SD calculated from three individual experiments. **f** HCO_3_ dehydration activity of COG4337 proteins and BCA

The widely conserved COG4337 proteins show partial sequence homology with a low CO_2_-inducible protein of the diatom *Thalassiosira pseudonana* (TpLCIP63), which was recently characterized as ι-class CA [4]. TpLCIP63 contains another bacterial conserved domain “COG4875” [20]. COG4337 and COG4875 domains are classified into different superfamilies, whereas they share a conserved short motif “His-His-Ser-Ser” in their C-termini (Additional file 2: Figure S2). On the basis of this observation, we investigated COG4337 proteins as a candidate CA. To perform the CA activity assay, two COG4337 proteins, Bn86287 and all2909, were selected from the eukaryotic microalga *Bigelowiella natans* and the cyanobacterium *Anabaena* sp. PCC7120, respectively, because complete genome sequences are available in these two organisms [21, 22]. Bn86287 consisted of three repeat domains, and all2909 had a single domain (Fig. 1a, b). We evaluated enzyme activity in Wilbur-Anderson units (WAU) [23] using recombinant proteins of Bn86287 (104–607 amino acids) and all2909 (34–206 amino acids) without N-terminal leaders. Both proteins showed significant CO_2_ hydration activity, with 85.8 ± 7.9 and 16.7 ± 0.5 WAU·mg^−1^ protein, respectively (Fig. 1c, d). The value of Bn86287 was within the range of those of the recombinant θ-CA from *Phaeodactylum tricornutum* (30.9 ± 0.8 WAU·mg^−1^ protein) [24] and the *T. pseudonana* ι-CA (122 ± 28 WAU·mg^−1^ protein) [4] and was lower compared to that of α-class bovine CA (BCA), approximately 600 WAU·mg^−1^ protein (Fig. 1e). We also calculated HCO_3_^-^ dehydration activity for Bn86287 and all2909, which showed very low values, 4 to 6 WAU·mg^−1^ protein (Fig. 1f). Some CAs are known to act as esterases on 4-nitrophenyl acetate [4, 24, 25]; however, neither Bn86287 nor all2909 exhibited obvious esterase activity (Additional file 2: Figure S3).

To assess the requirement of metal cofactors for catalysis, we tested the effects of chelating agents and metal ions on COG4337 proteins. The recombinant Bn86287 and all2909 proteins were treated with either 50 mM EDTA and 6 M urea or 50 mM 2,6-pyridinedicarboxylic acid (PDA) for 5 h, followed by dialysis against a metal-free buffer (20 mM Tris-HCl, 100 mM NaCl, 1 mM EDTA, pH 8.0). Unexpectedly, these treatments caused no decrease in CA activity (Fig. 1c, d), whereas the active site zinc ions of BCA were able to be removed by the same treatments (Fig. 1e). Next, Bn86287 and all2909 were treated with 2 mM Mg^2+^, Ca^2+^, Mn^2+^, Fe^2+^, Co^2+^, Ni^2+^, Zn^2+^, or Cd^2+^ to check whether the CA activity would be affected by the addition of divalent metal ions. Several metals caused partial precipitation of proteins, which was removed by centrifugation. The CA activity did not increase in all metal treatment groups, and the addition of zinc ions appears to negatively affect the enzyme activity in both Bn86287 and all2909 (Fig. 1c, d). To further support the metal independence of the COG4337 proteins, we performed an inductively coupled plasma optical emission spectroscopy (ICP-OES) analysis of the six metals (Mg, Ca, Mn, Co, Zn, and Cd). No such metals were found to bind to Bn86287 and all2909, whereas zinc was obtained in BCA at a predicted concentration (Additional file 3: Table S2). Taken together, these results suggested that the COG4337 proteins are metal-free enzymes that catalyze the hydration of CO_2_ to HCO_3_^-^, but not the reverse reaction.

### Overall structure

**Fig. 2:**
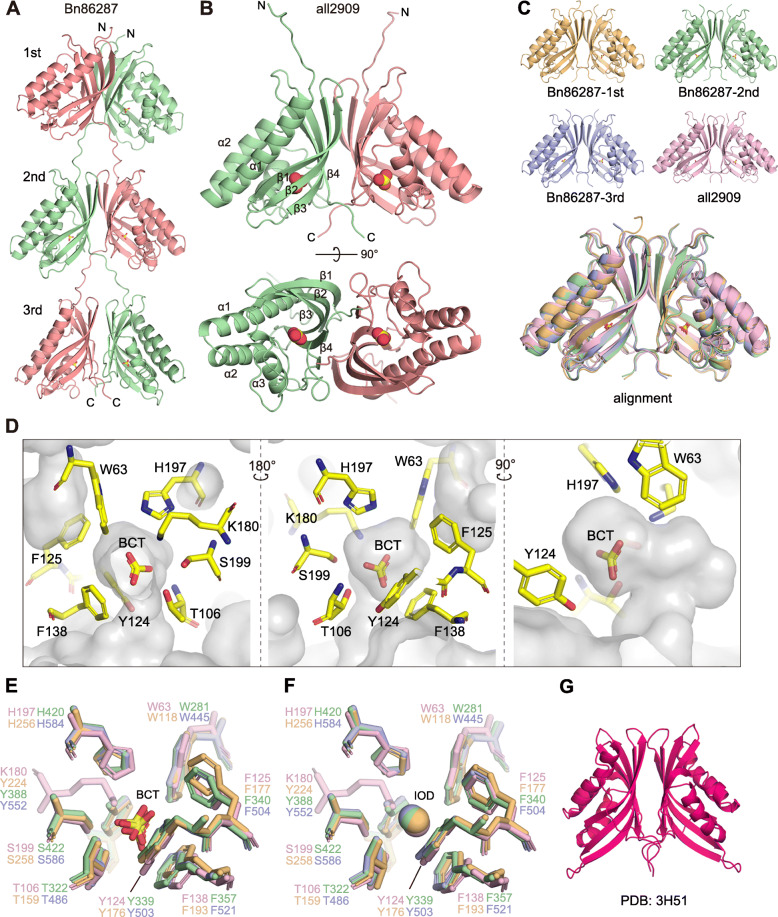
Structures of COG4337 proteins. **a**, **b** Dimeric structures of Bn86287 and all2909 are displayed as a ribbon diagram. A bicarbonate molecule (yellow and red spheres) is located inside the cone-shaped barrel. **c** Structural alignment of the four COG4337 domains. **d** Cross-sections of all2909 represent a bent finger-like cavity (transparent gay) containing a bicarbonate (BCT) and residues lining the cavity. **e**, **f** Active site overlays of the four COG4337 domains with bicarbonate (BCT) and iodide ion (IOD), respectively. As a difference, the position of Lys180 in all2909 is occupied by Tyr224/388/552 in Bn86287. **g** Dimeric structure of an uncharacterized protein of *Xanthomonas campestris* (PDB ID: 3H51). All images were prepared with PyMOL v. 2.3.3 (Schrödinger)

To further analyze the metal-free catalytic mechanism of COG4337 proteins, crystal structures of Bn86287 and all2909 were determined (Fig. 2, Table 1).

**Table 1:** Data collection and refinement statistics

	all2909		Bn86287	
	Bicarbonate complex	Iodide complex	Bicarbonate complex	Iodide complex
**Data collection**				
Space group	*P*2_1_2_1_2	*P*2_1_2_1_2	*P*2_1_	*P*2_1_
Cell dimensions				
*a*, *b*, *c* (Å)	52.8, 83.7, 87.3	53.5, 83.0, 88.1	46.9, 194.3, 58.3	46.8, 192.9, 58.2
*α*, *β*, *γ* (°)	90.0, 90.0, 90.0	90.0, 90.0, 90.0	90.0, 96.9, 90.0	90.0, 96.5, 90.0
Resolution (Å)	45.20–2.65	45.75–2.30	48.58–2.55	49.60–2.20
	(2.79–2.65)	(2.42–2.30)	(2.69–2.55)	(2.32–2.20)
*R*merge (%)	9.4 (69.4)	5.8 (90.0)	18.5 (39.3)	9.8 (32.0)
*I*/*σI*	12.49 (2.39)	25.18 (2.48)	8.4 (3.8)	14.6 (5.2)
Completeness (%)	100.00 (100.00)	99.90 (99.60)	99.8 (99.4)	99.0 (97.2)
Redundancy	7.0 (7.1)	13.6 (12.6)	7.0 (6.9)	7.0 (7.0)
**Refinement**				
Resolution (Å)	45.20–2.65	45.75–2.30	48.57–2.55	49.60–2.20
No. reflections	11758	18042	33627	51282
*R*work/*R*free (%)	20.7/26.3	22.1/24.4	19.2/24.3	16.7/20.7
No. of atoms				
Protein/ligand/ion/water	2401/8/–/5	2411/–/2/12	7500/24/–/206	7538/–/6/581
B-factors				
Protein/ligand/ion/water	58.8/64.4/–/51.7	64.1/–/64.4/56.6	18.2/22.3/–/13.4	22.5/–/19.8/25.2
R.m.s deviations				
Bond lengths (Å)	0.007	0.013	0.007	0.007
Bond angles (°)	0.917	1.688	0.877	0.756
Ramachandran plot				
Favored/allowed/outliers	95.0/5.0/0.0	96.3/3.7/0.0	96.0/4.0/0.0	97.4/2.6/0.0
PDB ID	7C5V	7C5W	7C5X	7C5Y

Each structure was determined from single-crystal diffraction. The highest resolution shell is shown in parentheses

Crystallographic analysis was performed using crystals soaked in solutions with bicarbonate and the anion inhibitor iodide; the addition of 1 mM KI led to the deactivation of the COG4337 proteins (Fig. 1c, d). Iodide ions have been reported as an inhibitor in several classes of CAs [26]. The crystal structures showed that both Bn86287 and all2909 seemed to form a homodimer (Fig. 2a, b). The results of size exclusion chromatography with multi-angle static light scattering (SEC-MALS) system showed that Bn86287 exists as dimers but all2909 exists as tetramers in solution (Additional file 2: Figure S4). Analysis with the PISA (Protein Interfaces, Surfaces and Assemblies) software [27] estimated that a tetramer of all2909 was assembled by a head-to-head interaction of two dimeric units (Additional file 2: Figure S4). Each domain formed a cone-shaped barrel structure comprising three α-helices and a four-stranded antiparallel β-sheet, which were almost identical across the COG4337 domains of Bn86287 and all2909 (Fig. 2c). This folding has no similarity with that of other CAs in α-, β-, γ-, ζ-, and θ-classes. However, DALI server searches [28] revealed that an uncharacterized protein of the γ-proteobacterium *Xanthomonas campestris* (PDB ID: 3H51) shares a similar fold to the COG4337 domains (Fig. 2g, Additional file 2: Figure S5) with a *z*-score of 15.2. Intriguingly, this uncharacterized protein exhibited 38 and 42% sequence identity with ι-class CAs of the diatom *T. pseudonana* and the bacterium *Burkholderia territorii*, respectively [29]. Although CA activity has not been confirmed in the *X. campestris* protein and no crystal structures are available for the *T. pseudonana* and *B. territorii* proteins, ι-CAs are potentially a structural homolog of COG4337 proteins.

### Catalytic active site

**Fig. 3:**
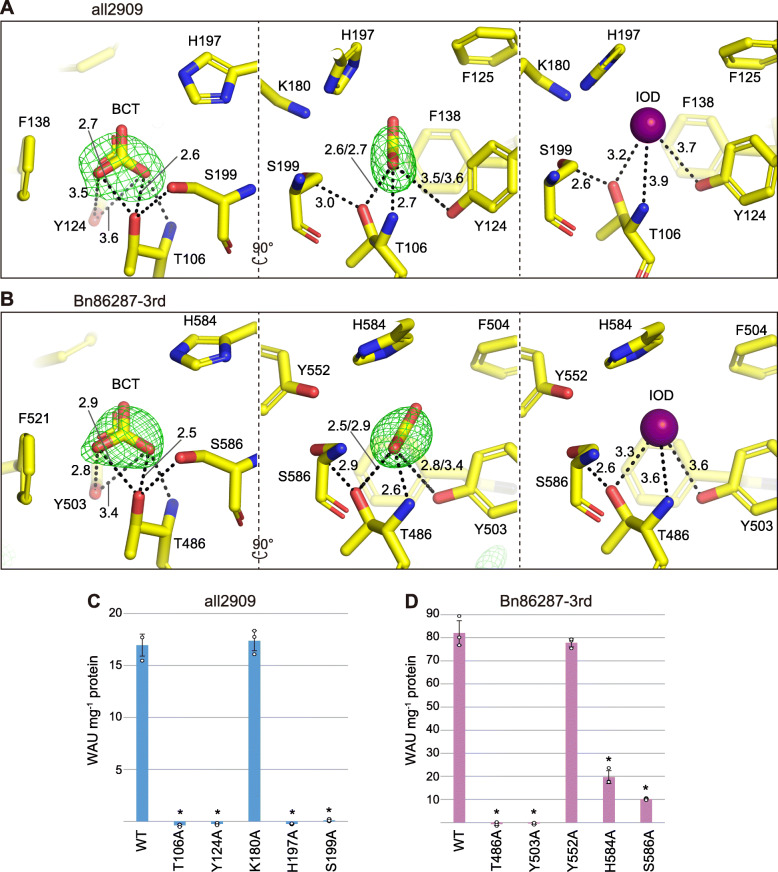
Point mutations of active site residues. **a**, **b** Active sites of all2909 and the 3rd domain of Bn86287. Simulated annealing *F*_o_–*F*_c_ omit maps (green) for bicarbonate (BCT) are displayed at a contour level of 3.0 *σ*. Relevant distances between bicarbonate oxygen atoms/iodide (IOD) and neighbor residues are indicated by dashed lines with numbers. The images were prepared with PyMOL v. 2.3.3 (Schrödinger). **c**, **d** CO_2_ hydration activity of all2909 and the 3rd domain of Bn86287 and their mutants. Mutated residues are indicated by the one-letter code and number. Error bars indicate the SD of triplicate experiments. Significant differences compared to the WT were determined by the two-tailed Student’s *t* test and indicated by asterisks (*P* < 0.005)

The substrate (bicarbonate) and inhibitor (iodide ion) were found in a bent finger-like cavity of the cone-shaped barrel (Fig. 2d). As expected from the biochemical assays described above, electron densities corresponding to metals were not detected in the cavity. Residues lining the cavity surface around bicarbonate/iodide were mostly conserved in the COG4337 domains (Fig. 2e, f), as well as in the apparent ι-CA of *X. campestris* (Additional file 2: Figure S5). One part of the cavity was dominated by hydrophilic residues (Thr, Ser, and His) and another consisted of hydrophobic residues (Trp and Phe). Notably, Lys180 in all2909 was found at the analogous position of Tyr224/388/552 in Bn86287 (Fig. 2e, f). To evaluate the functional importance of the cavity-forming residues, we performed point mutation analysis, wherein Thr106, Tyr124, Lys180, His197, and Ser199 were substituted by alanine in all2909 (Fig. 3a, c), and Thr486, Tyr503, Tyr552, His584, and Ser586 were replaced by alanine using a recombinant protein of the third COG4337 domain (431–607 amino acids) in Bn86287 (Fig. 3b, d). In all2909, the substitution of Lys180 did not affect the CA activity, while the other four mutations resulted in complete inactivation (Fig. 3c). In Bn8628, the Thr486 and Tyr503 mutants showed no CA activity, whereas the Tyr552 substitution had minimal effect on the activity (Fig. 3d). The mutations in residues His584 and Ser586 caused a 4- and 8-fold decrease in the CA activity compared to the wild type, respectively (Fig. 3d). These results suggested that an active site exists in the cavity, and Thr106/486 and Tyr124/503 residues (residue numbers in all2909/Bn86287) are necessary for enzyme catalysis. His197/584 and Ser199/586 were also determined to be important residues, whereas the non-conserved residues (i.e., Lys180 in all2909 and Tyr552 in Bn86287) in the cavity did not appear to be involved in the catalytic activity. By the way, it is worth noting that the third domain of Bn86287 exhibited a similar CA activity (82.1 ± 5.3 WAU·mg^−1^ protein) to the recombinant protein containing all three domains (Fig. 3d). It seems that multiple domains of Bn86287 do not work cooperatively, and each domain of Bn86287 would have a relatively high activity compared to the single domain of all2909.

### Putative catalytic mechanism

Based on the crystal structures and the results of point mutation analysis, we proposed a potential catalytic mechanism for CO_2_ hydration by COG4337 proteins. In other CAs, the initial step of the reaction involves the deprotonation of active site water to an OH^−^ ion, which further acts as a nucleophile and attacks CO_2_ to generate HCO_3_^-^ [2]. In an α-class human CA (hCAII), the Thr199-Glu106 network is assumed to accept a hydrogen bond from the zinc-bound water [10], and His64 mediates the proton transfer from the active site to bulk solvent [30]. Considering that hydroxyl groups of Thr106/159/322/486 (residue numbers in all2909/1st/2nd/3rd domain of Bn86287) and Tyr124/176/339/503 were found at a distance of 2.5 to 3.5 Å from an oxygen of HCO_3_^-^ and at a distance of 3.2 to 3.7 Å from an iodide ion (Fig. 3a, b, Additional file 2: Figure S5), these hydroxyl groups would most likely mediate the deprotonation of active site water. Similarly, an iodide inhibitor was reported to be positioned 3.6 Å from the hydroxyl group of Thr199 in hCAII [31]. The deprotonation process could also be assisted by the main chain nitrogen of Thr106/159/322/486 and the hydroxyl group of Ser199/258/422/586 that is positioned within hydrogen bond distance to Thr106/159/322/486. Although histidine is known to be a suitable proton-shuttle residue, His197/256/420/584 does not seem to serve this purpose, because it is located at the deep end of the cavity, far from the protein surface (Fig. 2d). The active site Tyr124/176/339/503 would likely acts as a proton-shuttle residue, as it faces the cavity and is proximal to the protein surface (Fig. 2d). Indeed, according to a previous report, an active site tyrosine in the β-CA of *Pisum sativum* mediates the proton transfer [32]. It has been reported that CO_2_ is located in a hydrophobic pocket near a phenylalanine in hCAII [33]. Assuming the same conformation for COG4337 proteins, CO_2_ might possibly be positioned toward the hydrophobic part near Phe138/193/357/521 (Fig. 3a, b, Additional file 2: Figure S5). However, further experiments are necessary to identify the CO_2_-binding site as well as the route of the proton from the active site to bulk solvent. Our analysis revealed that COG4337 proteins exhibited no obvious activity of HCO_3_^-^ dehydration (Fig. 1f), though other CAs are able to catalyze the reversible reaction of CO_2_ to HCO_3_^-^ [1, 2]. This peculiar feature might be related to the absence of metal in their active sites, but further study is required to assess this possibility.

### Subcellular localization

**Fig. 4:**
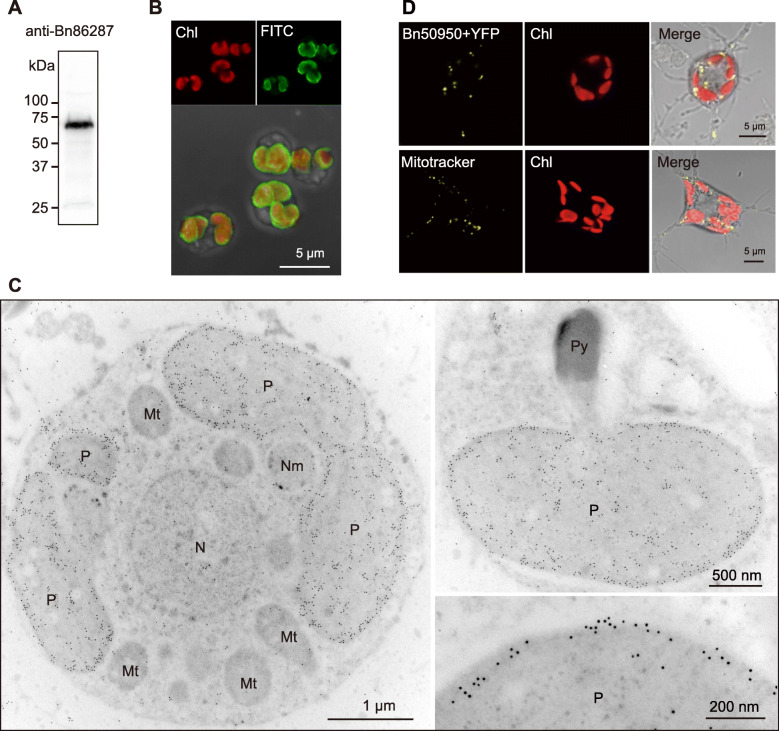
Subcellular localization of Bn86287 and Bn50950. **a** Immunoblot analysis of an anti-Bn86287 antibody against total proteins of *Bigelowiella natans*. A mature form of Bn86287 is estimated to be 55–59 kDa. **b** Confocal images of *B. natans* cells labeling with the anti-Bn86287 and an FITC-conjugated secondary antibody. Chlorophyll autofluorescence (Chl) is shown in red. **c** Immungold localization of Bn86287 in the plastid stroma of *B. natans*. Mt, mitochondrion; N, nucleus; Nm, nucleomorph; P, plastid; Py, pyrenoid. **d** Confocal images of the chlorarachniophyte *Amorphochlora amoebiformis* expressing the YFP fusion protein of mitochondrial Bn50950 (upper), and a cell stained by MitoTracker Orange CMTMRos (lower)

Next, we analyzed the localization of COG4337 proteins to elucidate their cellular functions. Bn86287 carries an N-terminal bipartite plastid targeting signal consisting of a signal peptide and transit peptide. Immunolocalization experiments demonstrated Bn86287 to be localized in the plastid stroma, accumulated at its periphery, but not in the pyrenoid (Fig. 4); the pyrenoid of *B. natans* was projected from the plastid stroma [34]. This localization pattern implicated Bn86287 to be involved partly in biophysical CO_2_-concentrating mechanisms (CCMs), whereby it possibly serves to recapture the unfixed CO_2_ leaking out of plastid stroma by CO_2_ hydration. Although several algal species are assumed to have a C_4_ photosynthesis pathway [35, 36], there is no evidence for such pathway in *B. natans*. Therefore, it remains unknown whether Bn86287 is involved in biochemical CCMs in which HCO_3_^-^ is fixed into C_4_ compounds, such as oxaloacetate. We found the *B. natans* genome to encode another COG4337 protein (Bn50950), consisting of a single COG4337 domain and a mitochondrial targeting signal (Fig. 4d). The mitochondrial COG4337 might be involved in buffering of the matrix pH and providing HCO_3_^-^ for anaplerotic reactions, as in mitochondrial β-CAs of *Chlamydomonas reinhardtii* [18]. We also performed in silico localization prediction for 62 eukaryotic COG4337 proteins and found 14 sequences to contain a putative plastid-targeting signal, and 19 sequences to carry a mitochondrial targeting peptide (Additional file 1: Table S1). Therefore, COG4337 proteins seemed to commonly function in plastids and mitochondria in various algae. On the contrary, most bacterial COG4337 proteins were predicted to carry a canonical N-terminal signal peptide, probably for their periplasmic or extracellular localization (Additional file 1: Table S1). They might play a role in CO_2_ hydration, for pH homeostasis and metabolic needs, which have been speculated for periplasmic α-CAs [37].

### Comparison between COG4337 and COG4875 proteins

As mentioned above, COG4337 proteins would potentially be a structural homolog of ι-class CAs carrying COG4875 domains, and both COG4337 and COG4875 domains shared a conserved C-terminal sequence motif “His-His-Ser-Ser.” Unlike COG4337 proteins, however, the ι-CA of *T. pseudonana* has been reported as a metalloenzyme containing Mn^2+^, based on the experiment that its metal-chelated protein was reactivated by the addition of Mn^2+^ [4], and the bacterial ι-CA of *B. territorii* has been speculated to bind Zn^2+^ [29, 38]. Metal-binding sites of ι-CAs remain unclear, and their active sites and catalytic mechanisms are also unknown. Interestingly, COG4337 proteins and ι-CAs shared several characteristics other than overall structure. The *T. pseudonana* ι-CA was reported to be localized at the periphery of plastid stroma [4], which resembles the localization of Bn86287. The diatom ι-CAs carried two to four repeat-domains as in eukaryotic COG4337 proteins [4]. In contrast, prokaryotic COG4875 and COG4337 proteins consisted of a single domain, and they both were predicted to possess an N-terminal signal peptide probably for periplasmic localization [29]. It was reported that transcription of the *T. pseudonana* ι-CA was strongly induced only under low CO_2_ conditions [20]. Although it remains unknown whether Bn86287 is regulated by CO_2_ conditions, the Bn86287 gene has been reported to be abundantly expressed with a diurnal rhythm [39]. Both COG4337 and COG4875 genes were widely found in bacteria and eukaryotic algae. However, these two genes very rarely coexist in an organism. For example, COG4337 and COG4875 genes were detected in thousands of genomes from diverse proteobacteria by BLAST searches, but surprisingly, only a few genomes carried both genes. These two genes are distributed in bacteria regardless of their phylogenetic positions, as even closely related species possessed either one. In cyanobacteria from the genus *Synechococcus*, strains CC9605 and WH7805 have a COG4337 gene, whereas strains CC9311 and WH8020 possess a COG4875 gene. Interestingly, CC9311 and WH8020 have been isolated from coastal environments, while CC9605 and WH7805 are open-ocean strains [40–42]. It seems reasonable to suppose that the COG4337-bearing cyanobacterial strains are adapted to low metal availability in the open ocean. Although it remains unclear why most organisms do not possess COG4337 and COG4875 genes together, it can be assumed that the coexistence of these two types of CAs may cause an unfavorable situation for cells.

## Conclusions

In this study, previously uncharacterized COG4337 proteins were confirmed to be CA enzymes catalyzing CO_2_ hydration. COG4337 proteins were found to be metal-free enzymes, unlike any known CAs. COG4337 proteins exhibited similarity to ι-CAs in sequence, overall structure, and some other characteristics, except that ι-CAs have been reported as a metalloenzyme. We thus concluded that COG4337 proteins should be treated as a new variant of ι-class CAs. At present, ι-CAs are able to divide into metal-free COG4337-type and metal-dependent COG4875-type. The property of COG4337-type ι-CAs would be an advantage to avoid competition with other metalloproteins for trace metals. In other words, they can function even in metal-poor environments (e.g., the open ocean); COG4337-type might have evolved in an ancestral prokaryote in response to such environment and subsequently have been inherited in various eukaryotic lineages. Considering the widespread prevalence of ι-class CAs across microalgae including ecologically important species [4, 19, 43], this class of CAs may play a role in the global carbon cycle.

## Methods

### Homology search and phylogenetic analysis

Homologous sequences containing COG4337 domains were detected by BLAST searches; prokaryotic homologs were searched by TBLASTN against the RefSeq genome database in NCBI (https://www.ncbi.nlm.nih.gov/) using all2909 of *Anabaena* sp. PCC 7120 (GenBank: BAB74608) as a query with an *E* value cutoff of 1e−20. Eukaryotic homologs were searched by BLASTP against protein sequences in NCBI, Joint Genome Institute (JGI) Genome Portal (https://genome.jgi.doe.gov/portal/), Phytozome v12.1 (https://phytozome.jgi.doe.gov/pz/portal.html), and the Marine Microbial Eukaryotic Transcriptome Sequencing Project (MMETSP) database [44] downloaded from iMicrobe (https://www.imicrobe.us/) using Bn86287 of *Bigelowiella natans* (JGI_Bignal: Protein ID 86287) [21] as a query with an *E* value cutoff of 1e−10. To perform phylogenetic analyses, 30 prokaryotic and 184 eukaryotic COG4337 domains were extracted from the collected homologs (Additional file 1: Table S1). Sequences were aligned by the L-INS-i method of the MAFFT package [45], and gaps and poorly aligned positions were removed by trimAl with the gappyout option [46]. Maximum likelihood trees were reconstructed using IQ-TREE [47] under the WAG+R7 model selected by ModelFinder [48] as the best-fitting model. Branch support values were evaluated with 100 standard non-parametric bootstrap replicates.

### Plasmid construction

Total RNA was extracted from *B. natans* (CCMP621) cells using TRIzol Reagent (Invitrogen), and cDNA was synthesized using SuperScript III Reverse Transcriptase (Invitrogen) with an oligo (dT) primer. Protein-coding sequences of Bn86287 and Bn50950 (JGI_Bignal: PROTEIN ID 86287 and 50950) were amplified with Ex Taq DNA polymerase (Takara) using the cDNA as a template and cloned into pGEM-T easy vectors (Promega). A double-stranded DNA encoding all2909 of *Anabaena* sp. PCC 7120 was synthetized de novo by Eurofins (Tokyo, Japan). Fragments coding Bn86287 (from 104 to 607/from 431 to 607 amino acids) and all2909 (from 31 to 205 amino acids) were amplified with KOD One PCR Master Mix and inserted into pET28a vectors (Novagen) between *Nde*I and *Eco*RI sites using GeneArt Seamless Cloning and Assembly Enzyme Mix (Invitrogen). The plasmids, named pET28-Bn86287:104-607, pET28-Bn86287:431-607, and pET28-all2909:31-205, were cloned into the DH5α strain of *Escherichia coli*. To generate point mutations (T106A, Y124A, K180A, H197A, and S199A in all2909, and T486A, Y503A, Y552A, H584A, and S586A in Bn86287), pET28-all2909:31-205 and pET28-Bn86287:431-607 were amplified with a set of outward-facing, overlapping primers containing nucleotide substitutions (Additional file 3: Table S3), and the products were self-assembled by GeneArt Seamless Cloning and Assembly Enzyme Mix (Invitrogen) and cloned into DH5α. All inserted sequences were verified by Sanger sequencing.

### Protein expression and purification

The pET28-derived plasmids were transformed into the Rosetta 2 (DE3) strain of *E. coli* (Novagen). To express recombinant proteins, the *E. coli* cells were grown in LB medium at 37°C, and isopropyl β-d-1-thiogalactopyranoside (IPTG) was added at OD_600_=0.5 to a final concentration of 1 mM. After 4 h of incubation at 37°C, the cells were harvested (approximately 1.0 g) and resuspended by 10 ml BugBuster Protein Extraction Reagent (Novagen) containing 150 units of Benzonase Nuclease (Novagen), 30,000 units of rLysozyme Solution (Novagen), and Complete Protease Inhibitor Cocktail (Roche). After removing the insoluble components by centrifugation, recombinant proteins in supernatants were purified with His GraviTrap columns (Cytiva), according to the manufacturer’s instruction. The eluted protein solution was replaced by a buffer containing 20 mM Tris-HCl (pH 8.0) and 100 mM NaCl using PD10 desalting columns (Cytiva). To remove an N-terminal His-tag, the protein solution (approximately 1 mg/ml) was treated with thrombin protease (Novagen) at a concentration of 0.5–2 units/ml for 12 h at 20°C. For crystallization, recombinant proteins were concentrated to 7–10 mg/ml using an Amicon Ultra-4 centrifugal filter (10 K and 30 K MWCO were used for all2909 and Bn86287, respectively). Protein concentration was determined using the Qubit Protein Assay kit with a Qubit 3.0 fluorometer (Thermo Fisher Scientific). All purified protein samples were stored at 4°C prior to analysis.

### Enzymatic assays

CA activity was measured using the Wilbur and Anderson method [23] along with some modifications [24]. CO_2_ hydration reaction was monitored by the drop in pH from 8.3 to 7.8 when 4 ml of ice-cold CO_2_ saturated water was added into 6 ml of ice-cold 20 mM Tris-H_2_SO_4_ (pH 8.3 at 20°C), with or without 6–40 μg protein. Alternatively, HCO_3_^-^ dehydration reaction was monitored by the rise in pH from 5.6 to 5.9 when 4 ml of ice-cold 50 mM NaHCO_3_ was added into 6 ml of ice-cold 50 mM MES-NaOH (pH 5.3 at 20°C), with or without 10–100 μg protein. CA activity was calculated in Wilbur and Anderson units (WAU) mg^−1^ protein according to the following equation: WAU = *T*_0_/*T*_1_−1, where *T*_1_ is the time for pH change in presence of proteins and *T*_0_ is the time in the absence of proteins [23]. Bovine erythrocyte CA (BCA, Sigma-Aldrich, C3934) and bovine serum albumin (BSA, Sigma-Aldrich, A9647) were used as positive and negative controls, respectively. For the chelation of protein-binding metal ions, proteins were treated either with buffer A (50 mM EDTA and 6 M urea in 20 mM Tris-HCl, pH 8.0) or buffer B (50 mM 2,6-pyridinedicarboxylic acid, 12.5 mM MOPS, pH 7.0) for 5 h at 20°C, followed by overnight dialysis against 20 mM Tris-HCl (pH 8.0), 100 mM NaCl, and 1 mM EDTA in Slide-A-Lyzer MINI 10K Device (Thermo Fisher Scientific). These two chelating buffers were selected based on previous studies [49, 50]. Reactivation of apo-BCA was achieved by the addition of 2 mM ZnCl_2_. To test the effects of divalent metals on the activity of all2909 and Bn86287, 2 mM MnCl_2_, MgCl_2_, CaCl_2_, CoCl_2_, NiCl_2_, ZnCl_2_ FeCl_2_, or CdCl_2_ was added to the non-treated proteins and incubated for 2 h at room temperature. Several metal treatments (i.e., all2909+Co, all2909+Zn, all2909+Cd, all2909+Fe, Bn86287+Zn, Bn86287+Cd, and Bn86287+Fe) caused partial protein precipitation, which was removed by centrifugation. Esterase activity was determined using 4-nitrophenyl acetate (Sigma-Aldrich), as described previously [51]. We measured the increase in absorption at 348 nm for 5 min at room temperature after the addition of 40 μg protein to a reaction mixture containing 0.3 ml of 3 mM 4-nitrophenyl acetate and 0.7 ml of 20 mM Tris-HCl (pH 8.0).

### ICP-OES

To remove free metals, BCA (Sigma-Aldrich, C3934) and the recombinant proteins Bn86287 and all2909 were dialyzed against a buffer containing 20 mM Tris-HCl (pH 8.0) and 100 mM NaCl in Slide-A-Lyzer MINI 10K Device (Thermo Fisher Scientific). The concentrations of resulting protein solutions were estimated to be 1.1 to 1.4 mg/ml. Each protein solution was diluted 50-fold with 10 ml of Milli-Q water, and the same amount of solution was injected into the spray chamber in the Optima 2100 DV ICP-OES (PerkinElmer). The mass percentages of the six metals (Mg, Ca, Mn, Co, Zn, and Cd) were obtained for each protein solution.

### Crystallization

Crystallization conditions were initially screened using Crystal Screen 1 and 2 (Hampton Research), Wizard Screens I and II (Rigaku), PEGsII (Qiagen), Index (Hampton Research), PEGIon/PEGIon2 (Hampton Research), and a Protein Complex Suite (Qiagen) with a Protein Crystallization System (PXS) at the Structural Biology Research Center, High Energy Accelerator Research Organization in Japan [52]. Screening was performed by the sitting-drop vapor-diffusion method with crystallization drops consisting of 0.2 μl protein solutions (7.0 mg/ml) and 0.2 μl screening solutions at 293 K and 277 K. Crystals of all2909 were observed after 1 week under the conditions of Index #27 (2.4 M sodium malonate, pH 7.0) at 293 K. Before diffraction data collection, crystals of all2909 were cryoprotected in a solution containing 30% glycerol and 1.7 M sodium malonate, pH 7.0, for 30 s. For the iodide-SAD phasing, crystals were soaked in iodide-containing artificial mother liquor (25 mM KI and 2.4 M sodium malonate, pH 7.0) for 1.5 h, and then cryoprotected in 30% glycerol solution with 25 mM KI and 1.7 M sodium malonate, pH 7.0, for 30 s. Crystals of the all2909-HCO_3_^-^ complex were prepared by soaking crystals into the solution containing 50 mM NaHCO_3_, 30% glycerol, and 1.7 M sodium malonate, pH 7.0, for 30 s. Crystals of Bn86287 were observed after 1 month under the conditions of Protein Complex #31 (20% PEG4000, 20% 2-propanol, and 0.1 M sodium citrate, pH 5.6) at 277 K. Before diffraction data collection, crystals of Bn86287 were cryoprotected in a solution containing 30% glycerol, 14% PEG4000, 14% 2-propanol, and 70 mM sodium citrate, pH 5.6, for 15 s. Crystals of the Bn86287-HCO_3_^-^ complex were prepared by soaking crystals in NaHCO_3_-containing solution supplemented with 30% glycerol, 14% PEG4000, 14% 2-propanol, and 70 mM sodium citrate, pH 5.6, for 2 min.

### Data collection and structure determination

X-ray diffraction data were collected at 95 K using an Eiger X4M detector on BL-1A, or an Eiger X16M detector on BL-17A, of the Photon Factory, KEK (Tsukuba, Japan). Diffraction data were processed and scaled by XDS and XSCALE, respectively [53]. The phases of all2909 were determined using the program Crank2 by the iodide-SAD method [54]. The phases of Bn86287 were determined using an MR-native SAD method. The coordinates of all2909 were used as the initial model for MR calculation by MOLREP [55], and the obtained initial phases were used for the MR-native SAD calculation by Crank2. Crystallographic refinements and model building were performed using PHENIX.refine [56] and Coot [57], respectively.

### SEC-MALS

Size exclusion chromatography was performed with a Superdex 200 increase 10/300 GL column (Cytiva) using an Alliance 2695 system (Waters). Light scattering (LS) and refractive index (RI) were measured using a DAWN HELEOS II detector (Wyatt Technology) and 2414 RI detector (Waters), respectively. Before SEC-MALS analysis, the column was equilibrated at 293 K with 20 mM Tris-HCl, pH 8.0, containing 100 mM NaCl. Bn86287 (3.7 mg/ml, 30 μl) and all2909 (4.9 mg/ml, 30 μl) were injected under the buffer flow rate of 0.5 ml/min. Data were processed with the ASTRA 6.1 software (Wyatt Technology).

### Immunoblotting and immunolocalization

Polyclonal antibodies against Bn86287 were raised in rabbits using a recombinant protein corresponding to its third COG4337 domain (from 431 to 607 amino acids) by Kiwa Laboratory Animals. Co., Ltd. (Wakayama, Japan). The specificity of antibodies was tested by immunoblot analysis using total proteins of *B. natans*. The proteins were electrophoresed on an Any kD Mini-PROTEAN TGX gel (Bio-Rad) and blotted to a polyvinylidene difluoride membrane using a Trans-Blot Turbo Transfer System (Bio-Rad). Immunoblotting was performed using an iBind Western system (Life Technologies) with an anti-Bn86287 antibody diluted at 1:500, followed by a horseradish peroxidase (HRP)-linked secondary antibody (Cytiva, NA934VS, Lot#9790787) at a dilution of 1:10,000. The signals were detected with ECL Prime Western Blotting Detection Reagent (Cytiva) and a ChemiDoc MP System (Bio-Rad). Uncropped immunoblotting images are shown in Additional file 2: Figure S6. Immunofluorescence and immunoelectron microscopic analyses were performed according to the protocol described previously [58]. For immunofluorescence labeling, fixed *B. natans* cells were treated with a 1:100 dilution of anti-Bn86287 antibody and a fluorescein isothiocyanate (FITC)-conjugated secondary antibody (Sigma-Aldrich, F9887, Lot#108M4818V) diluted to 1:100. Fluorescence signals were observed under an inverted Zeiss LSM 510 laser scanning microscope (Carl Zeiss). For immunoelectron microscopy, immunogold labeling was performed with the anti-Bn86287 antibody at a dilution of 1:20 and a gold-conjugated secondary antibody (Sigma-Aldrich, G7402, Lot#SLBG4607V) diluted to 1:20. Labeled sections were stained with uranyl acetate and observed under a Hitachi H7650 transmission electron microscope at 80 kV.

### Subcellular localization prediction

We predicted the subcellular localization of 23 bacterial and 62 eukaryotic COG4337 homologs that were used for phylogenetic analysis. Partial sequences were removed from the prediction, based on an alignment with all homologs. The prediction of an N-terminal signal was conducted in four programs (PredSL [59], TargetP-2.0 [60], Predotar [61], and SignalP-5.0 [62]). For prasinophytes and rhodophytes, a “plant” setting was applied. In complex plastid-bearing algae (chlorarachniophytes, dinoflagellates, haptophytes, and ochrophytes), plastid-targeted proteins carry an N-terminal bipartite signal containing a signal peptide and transit peptide [63]. Transit peptides have been characterized by possessing positively charged residues in chlorarachniophytes [64] and an aromatic residue at the first position in dinoflagellates, haptophytes, and ochrophytes [63]. These characteristics were used to evaluate their plastid-targeting signals.

### Localization of GFP fusion protein

For plasmid construction, a fragment encoding the N-terminal leader of Bn50950 (from 1 to 38 amino acids) was inserted at the 5′ end of *yfp* gene in pLaRYfp+mc vector. The chlorarachniophyte species *Amorphochlora amoebiformis* (CCMP2058) was transfected with this plasmid using a Gene Pulser Xcell electroporation system (Bio-Rad), as described previously [65, 66], as no transformation system was available for *B. natans*. The mitochondria were stained with MitoTracker Orange CMTMRos (molecular probes) at a final concentration of 1 μM. Fluorescence signals were observed under an inverted Zeiss LSM 510 laser scanning microscope (Carl Zeiss).

## Supplementary Information

**Additional file 1: Table S1.** COG4337 proteins of diverse eukaryotes and prokaryotes.

**Additional file 2: Figure S1.** Maximum-likelihood phylogenetic tree of COG4337 proteins. The tree was contracted with 214 of COG4337 domain sequences extracted from 30 prokaryotic and 102 eukaryotic proteins. Sequences of multiple repeated domains are labelled by the ordinal number. Numbers at nodes indicate bootstrap supports (BS) that are shown only when they are higher than 50%. Black dots correspond to ≥95% BS. The scale bar represents the expected number of amino acid substitutions per site. **Figure S2.** Sequence alignment of COG4337 and COG4875 domains. The alignment includes COG4337 domains extracted from Bn86287 (JGI Bigna1: 86287), Bn50950 (JGI Bigna1: 50950), and all2909 (GenBank: BAB74608), and COG4875 domains of LCIP63 (JGI Thaps3: 9854) and 3H51 (GenBank: AAM40142). Numbers next to protein names represent the position of repeated domains. Asterisks show conserved amino acids, and the C-terminal motif “His-His-Ser-Ser” is highlighted by a yellow box. **Figure S3.** Esterase activity. Esterase activity was measured with 4-nitrophenyl acetate as substrate. Absorption at 348 nm was monitored for 5 min after the addition of each protein at the time point 60 sec. Values of esterase activity are summarized in the table (mean ± SD of three independent experiments). BSA, bovine serum albumin. **Figure S4.** SEC-MALS analysis of recombinant COG4337 proteins. (**A**, **B**) Light scattering (LS, red line), differential reflective index (dRI, blue line), and the molecular weight of the protein (black line) are plotted against the elution volume. Theoretical molar mass of the Bn82787 and all2909 monomer being 55.3 kDa and 19.3 kDa, respectively. Bn86287 and all2909 were estimated to exist as dimers and tetramers in solution, respectively. (**C**) Analysis with the PISA (Protein Interfaces, Surfaces and Assemblies) software estimated that a tetramer of all2909 was assembled by a head-to-head interaction of two dimeric units. **Figure S5.** Structural comparison of COG4337 domains. (**A**) Structural alignment of the all2909 (dark pink) and an uncharacterized protein of *Xanthomonas campestris* (PDB ID: 3H51) (dark pink). (**B**) Cavities of all2909 and 3H51 are constructed by almost identical residues. (**C, D**) Active sites of the 1st and 2nd COG4337 domain in Bn86287. Simulated annealing F_o_-F_c_ omit maps (green) for bicarbonate (BCT) are displayed at a contour level of 3.0 σ. Relevant distances between bicarbonate oxygen atoms/iodide (IOD) and neighbor residues are indicated by dashed lines with numbers. All images were prepared with PyMOL v. 2.3.3 (Schrödinger). **Figure S6.** Uncropped immunoblotting images. **(A)** Western blots against total proteins and the recombinant Bn86287 protein. The square shows the cropped region for Fig 4a. **(B)** Brightfield image of the membrane.

**Additional file 3: Table S2.** ICP-OES (inductively coupled plasma optical emission spectroscopy) analysis of six metals. **Table S3.** Primer sequences for plastid construction.

## Data Availability

Coordinates and structure factors have been deposited to the Protein Data Bank (PDB) under accession codes 7C5V (bicarbonate complex of all2909) [67], 7C5W (iodide complex of all2909) [68], 7C5X (bicarbonate complex of Bn86287) [69], and 7C5Y (iodide complex of Bn86287) [70]. The accession numbers for the sequences used in our phylogenetic analysis are given in Additional file 1: Table S1.

## References

[1] Tripp BC, Smith K, Ferry JG (2001). Carbonic anhydrase: new insights for an ancient enzyme. J Biol Chem.

[2] Supuran CT (2016). Structure and function of carbonic anhydrases. Biochem J.

[3] DiMario RJ, Machingura MC, Waldrop GL, Moroney JV (2018). The many types of carbonic anhydrases in photosynthetic organisms. Plant Sci.

[4] Jensen EL, Clement R, Kosta A, Maberly SC, Gontero B (2019). A new widespread subclass of carbonic anhydrase in marine phytoplankton. ISME J.

[5] Jin S, Sun J, Wunder T, Tang D, Cousins AB, Sze SK, Mueller-Cajar O, Gao YG (2016). Structural insights into the LCIB protein family reveals a new group of β-carbonic anhydrases. Proc Natl Acad Sci.

[6] Jin S, Vullo D, Bua S, Nocentini A, Supuran CT, Gao YG (2020). Structural and biochemical characterization of novel carbonic anhydrases from *Phaeodactylum tricornutum*. Acta Crystallogr Sect D Struct Biol.

[7] Del Prete S, Vullo D, Fisher GM, Andrews KT, Poulsen SA, Capasso C (2014). Discovery of a new family of carbonic anhydrases in the malaria pathogen *Plasmodium falciparum* - the η-carbonic anhydrases. Bioorg Med Chem Lett.

[8] Lane TW, Saito MA, George GN, Pickering IJ, Prince RC, Morel FMM (2005). A cadmium enzyme from a marine diatom. Nature..

[9] MacAuley SR, Zimmerman SA, Apolinario EE, Evilia C, Hou YM, Ferry JG (2009). The archetype γ-class carbonic anhydrase (cam) contains iron when synthesized in vivo. Biochemistry..

[10] Lindskog S (1997). Structure and mechanism of carbonic anhydrase. Pharmacol Ther.

[11] Cox EH, McLendon GL, Morel FMM, Lane TW, Prince RC, Pickering IJ (2000). The active site structure of *Thalassiosira weissflogii* carbonic anhydrase 1. Biochemistry..

[12] De Simone G, Di Fiore A, Capasso C, Supuran CT (2015). The zinc coordination pattern in the η-carbonic anhydrase from *Plasmodium falciparum* is different from all other carbonic anhydrase genetic families. Bioorg Med Chem Lett.

[13] Mitsuhashi S, Mizushima T, Yamashita E, Yamamoto M, Kumasaka T, Moriyama H, Ueki T, Miyachi S, Tsukihara T (2000). X-ray structure of β-carbonic anhydrase from the red alga, *Porphyridium purpureum*, reveals a novel catalytic site for CO_2_ hydration. J Biol Chem.

[14] Xu Y, Feng L, Jeffrey PD, Shi Y, Morel FMM (2008). Structure and metal exchange in the cadmium carbonic anhydrase of marine diatoms. Nature..

[15] Henry RP (1996). Multiple roles of carbonic anhydrase in cellular transport and metabolism. Annu Rev Physiol.

[16] Smith KS, Ferry JG (2000). Prokaryotic carbonic anhydrases. FEMS Microbiol Rev.

[17] DiMario RJ, Clayton H, Mukherjee A, Ludwig M, Moroney JV (2017). Plant carbonic anhydrases: structures, locations, evolution, and physiological roles. Mol Plant.

[18] Aspatwar A, Haapanen S, Parkkila S (2018). An update on the metabolic roles of carbonic anhydrases in the model alga *Chlamydomonas reinhardtii*. Metabolites..

[19] Leggat W, Hoegh-Guldberg O, Dove S, Yellowlees D (2007). Analysis of an EST library from the dinoflagellate (*Symbiodinium* sp.) symbiont of reef-building corals. J Phycol.

[20] Clement R, Lignon S, Mansuelle P, Jensen E, Pophillat M, Lebrun R, Denis Y, Puppo C, Maberly SC, Gontero B (2017). Responses of the marine diatom *Thalassiosira pseudonana* to changes in CO_2_ concentration: a proteomic approach. Sci Rep.

[21] Curtis BA, Tanifuji G, Burki F, Gruber A, Irimia M, Maruyama S, Arias MC, Ball SG, Gile GH, Hirakawa Y, Hopkins JF, Kuo A, Rensing SA, Schmutz J, Symeonidi A, Elias M, Eveleigh RJM, Herman EK, Klute MJ, Nakayama T, Oborník M, Reyes-Prieto A, Armbrust EV, Aves SJ, Beiko RG, Coutinho P, Dacks JB, Durnford DG, Fast NM, Green BR, Grisdale CJ, Hempel F, Henrissat B, Höppner MP, Ishida KI, Kim E, Kořený L, Kroth PG, Liu Y, Malik SB, Maier UG, McRose D, Mock T, Neilson JAD, Onodera NT, Poole AM, Pritham EJ, Richards TA, Rocap G, Roy SW, Sarai C, Schaack S, Shirato S, Slamovits CH, Spencer DF, Suzuki S, Worden AZ, Zauner S, Barry K, Bell C, Bharti AK, Crow JA, Grimwood J, Kramer R, Lindquist E, Lucas S, Salamov A, McFadden GI, Lane CE, Keeling PJ, Gray MW, Grigoriev IV, Archibald JM (2012). Algal genomes reveal evolutionary mosaicism and the fate of nucleomorphs. Nature..

[22] Kaneko T, Nakamura Y, Wolk CP, Kuritz T, Sasamoto S, Watanabe A, Iriguchi M, Ishikawa A, Kawashima K, Kimura T, Kishida Y, Kohara M, Matsumoto M, Matsuno A, Muraki A, Nakazaki N, Shimpo S, Sugimoto M, Takazawa M, Yamada M, Yasuda M, Tabata S (2001). Complete genomic sequence of the filamentous nitrogen-fixing cyanobacterium *Anabaena* sp. Strain PCC 7120. DNA Res.

[23] Wilbur KM, Anderson NG (1948). Electrometric and colorimetric determination of carbonic anhydrase. J Biol Chem.

[24] Kikutani S, Nakajima K, Nagasato C, Tsuji Y, Miyatake A, Matsuda Y (2016). Thylakoid luminal θ-carbonic anhydrase critical for growth and photosynthesis in the marine diatom *Phaeodactylum tricornutum*. Proc Natl Acad Sci.

[25] Verpoorte JA, Mehta S, Edsall JT (1967). Esterase activities of human carbonic anhydrases B and C. J Biol Chem.

[26] De Simone G, Supuran CT (2012). (In)organic anions as carbonic anhydrase inhibitors. J Inorg Biochem.

[27] Krissinel E, Henrick K (2007). Inference of macromolecular assemblies from crystalline state. J Mol Biol.

[28] Holm L (2020). DALI and the persistence of protein shape. Protein Sci.

[29] Del Prete S, Nocentini A, Supuran CT, Capasso C (2020). Bacterial ι-carbonic anhydrase: a new active class of carbonic anhydrase identified in the genome of the Gram-negative bacterium *Burkholderia territorii*. J Enzyme Inhib Med Chem.

[30] Silverman DN, Mckenna R (2007). Solvent-mediated proton transfer in catalysis by carbonic anhydrase. Acc Chem Res.

[31] Kumar V, Kannan KK, Sathyamurthi P (1994). Differences in anionic inhibition of human carbonic anhydrase I revealed from the structures of iodide and gold cyanide inhibitor complexes. Acta Crystallogr Sect D Biol Crystallogr.

[32] Kimber MS, Pai EF (2000). The active site architecture of *Pisum sativum* β-carbonic anhydrase is a mirror image of that of α-carbonic anhydrases. EMBO J.

[33] Kim CU, Song H, Avvaru BS, Gruner SM, Park S, McKenna R (2016). Tracking solvent and protein movement during CO_2_ release in carbonic anhydrase II crystals. Proc Natl Acad Sci U S A.

[34] Moestrup Ø, Sengco M (2001). Ultrastructural studies on *Bigelowiella natans*, gen. et sp. nov., a chlorarachniophyte flagellate. J Phycol.

[35] Matsuda Y, Nakajima K, Tachibana M (2011). Recent progresses on the genetic basis of the regulation of CO_2_ acquisition systems in response to CO_2_ concentration. Photosynth Res.

[36] Jensen EL, Maberly SC, Gontero B (2020). Insights on the functions and ecophysiological relevance of the diverse carbonic anhydrases in microalgae. Int J Mol Sci.

[37] Supuran C, Capasso C (2017). An overview of the bacterial carbonic anhydrases. Metabolites..

[38] Petreni A, De Luca V, Scaloni A, Nocentini A, Capasso C, Supuran CT (2021). Anion inhibition studies of the Zn(II)-bound ι-carbonic anhydrase from the Gram-negative bacterium *Burkholderia territorii*. J Enzyme Inhib Med Chem.

[39] Suzuki S, Ishida K, Hirakawa Y (2016). Diurnal transcriptional regulation of endosymbiotically derived genes in the chlorarachniophyte *Bigelowiella natans*. Genome Biol Evol.

[40] Fuller NJ, Marie D, Partensky F, Vaulot D, Post AF, Scanlan DJ (2003). Clade-specific 16S ribosomal DNA oligonucleotides reveal the predominance of a single marine *Synechococcus* clade throughout a stratified water column in the red sea. Appl Environ Microbiol.

[41] Palenik B, Ren Q, Dupont CL, Myers GS, Heidelberg JF, Badger JH, Madupu R, Nelson WC, Brinkac LM, Dodson RJ, Durkin AS, Daugherty SC, Sullivan SA, Khouri H, Mohamoud Y, Halpin R, Paulsen IT (2006). Genome sequence of *Synechococcus* CC9311: insights into adaptation to a coastal environment. Proc Natl Acad Sci U S A.

[42] Mackey KRM, Post AF, McIlvin MR, Cutter GA, John SG, Saito MA (2015). Divergent responses of Atlantic coastal and oceanic *Synechococcus* to iron limitation. Proc Natl Acad Sci U S A.

[43] Falkowski PG (2004). The evolution of modern eukaryotic phytoplankton. Science.

[44] Keeling PJ, Burki F, Wilcox HM, Allam B, Allen EE, Amaral-Zettler LA, Armbrust EV, Archibald JM, Bharti AK, Bell CJ, Beszteri B, Bidle KD, Cameron CT, Campbell L, Caron DA, Cattolico RA, Collier JL, Coyne K, Davy SK, Deschamps P, Dyhrman ST, Edvardsen B, Gates RD, Gobler CJ, Greenwood SJ, Guida SM, Jacobi JL, Jakobsen KS, James ER, Jenkins B, John U, Johnson MD, Juhl AR, Kamp A, Katz LA, Kiene R, Kudryavtsev A, Leander BS, Lin S, Lovejoy C, Lynn D, Marchetti A, McManus G, Nedelcu AM, Menden-Deuer S, Miceli C, Mock T, Montresor M, Moran MA, Murray S, Nadathur G, Nagai S, Ngam PB, Palenik B, Pawlowski J, Petroni G, Piganeau G, Posewitz MC, Rengefors K, Romano G, Rumpho ME, Rynearson T, Schilling KB, Schroeder DC, Simpson AGB, Slamovits CH, Smith DR, Smith GJ, Smith SR, Sosik HM, Stief P, Theriot E, Twary SN, Umale PE, Vaulot D, Wawrik B, Wheeler GL, Wilson WH, Xu Y, Zingone A, Worden AZ (2014). The Marine Microbial Eukaryote Transcriptome Sequencing Project (MMETSP): illuminating the functional diversity of eukaryotic life in the oceans through transcriptome sequencing. PLoS Biol.

[45] Katoh K, Standley DM (2013). MAFFT multiple sequence alignment software version 7: improvements in performance and usability. Mol Biol Evol.

[46] Capella-Gutiérrez S, Silla-Martínez JM, Gabaldón T (2009). trimAl: a tool for automated alignment trimming in large-scale phylogenetic analyses. Bioinformatics.

[47] Nguyen L-T, Schmidt HA, von Haeseler A, Minh BQ (2015). IQ-TREE: a fast and effective stochastic algorithm for estimating maximum-likelihood phylogenies. Mol Biol Evol.

[48] Kalyaanamoorthy S, Minh BQ, Wong TKF, Von Haeseler A, Jermiin LS (2017). ModelFinder: fast model selection for accurate phylogenetic estimates. Nat Methods.

[49] Lindskog S, Malmstrom BG (1962). Metal binding and catalytic activity in bovine carbonic anhydrase. J Biol Chem.

[50] Hunt JB, Rhee MJ, Storm CB (1977). A rapid and convenient preparation of apocarbonic anhydrase. Anal Biochem.

[51] Del Prete S, Vullo D, De Luca V, Supuran CT, Capasso C (2014). Biochemical characterization of the δ-carbonic anhydrase from the marine diatom *Thalassiosira weissflogii*, TweCA. J Enzyme Inhib Med Chem.

[52] Kato R, Hiraki M, Yamada Y, Tanabe M, Senda T (2021). A fully automated crystallization apparatus for small protein quantities. Acta Crystallogr Sect F Struct Biol Commun.

[53] Kabsch W (1993). Automatic processing of rotation diffraction data from crystals of initially unknown symmetry land cell constants. J Appl Crystallogr.

[54] Skubák P, Pannu NS (2013). Automatic protein structure solution from weak X-ray data. Nat Commun.

[55] Vagin A, Teplyakov A (1997). MOLREP: an automated program for molecular replacement. J Appl Crystallogr.

[56] Liebschner D, Afonine PV, Baker ML, Bunkoczi G, Chen VB, Croll TI (2019). Macromolecular structure determination using X-rays, neutrons and electrons: recent developments in Phenix. Acta Crystallogr Sect D Struct Biol.

[57] Emsley P, Cowtan K (2004). Coot: model-building tools for molecular graphics. Acta Crystallogr Sect D Biol Crystallogr.

[58] Hirakawa Y, Ishida K (2015). Prospective function of FtsZ proteins in the secondary plastid of chlorarachniophyte algae. BMC Plant Biol.

[59] Petsalaki EI, Bagos PG, Litou ZI, Hamodrakas SJ (2006). PredSL: a tool for the N-terminal sequence-based prediction of protein subcellular localization. Genomics Proteomics Bioinformatics.

[60] Almagro Armenteros JJ, Salvatore M, Emanuelsson O, Winther O, von Heijne G, Elofsson A (2019). Detecting sequence signals in targeting peptides using deep learning. Life Sci Alliance.

[61] Small I, Peeters N, Legeai F, Lurin C (2004). Predotar: a tool for rapidly screening proteomes for N-terminal targeting sequences. Proteomics..

[62] Almagro Armenteros JJ, Tsirigos KD, Sønderby CK, Petersen TN, Winther O, Brunak S, von Heijne G, Nielsen H (2019). SignalP 5.0 improves signal peptide predictions using deep neural networks. Nat Biotechnol.

[63] Patron NJ, Waller RF (2007). Transit peptide diversity and divergence: a global analysis of plastid targeting signals. BioEssays..

[64] Hirakawa Y, Gile GH, Ota S, Keeling PJ, Ishida K (2010). Characterization of periplastidal compartment-targeting signals in chlorarachniophytes. Mol Biol Evol.

[65] Faktorová D, Nisbet RER, Fernández Robledo JA, Casacuberta E, Sudek L, Allen AE, Ares M, Aresté C, Balestreri C, Barbrook AC, Beardslee P, Bender S, Booth DS, Bouget FY, Bowler C, Breglia SA, Brownlee C, Burger G, Cerutti H, Cesaroni R, Chiurillo MA, Clemente T, Coles DB, Collier JL, Cooney EC, Coyne K, Docampo R, Dupont CL, Edgcomb V, Einarsson E, Elustondo PA, Federici F, Freire-Beneitez V, Freyria NJ, Fukuda K, García PA, Girguis PR, Gomaa F, Gornik SG, Guo J, Hampl V, Hanawa Y, Haro-Contreras ER, Hehenberger E, Highfield A, Hirakawa Y, Hopes A, Howe CJ, Hu I, Ibañez J, Irwin NAT, Ishii Y, Janowicz NE, Jones AC, Kachale A, Fujimura-Kamada K, Kaur B, Kaye JZ, Kazana E, Keeling PJ, King N, Klobutcher LA, Lander N, Lassadi I, Li Z, Lin S, Lozano JC, Luan F, Maruyama S, Matute T, Miceli C, Minagawa J, Moosburner M, Najle SR, Nanjappa D, Nimmo IC, Noble L, Novák Vanclová AMG, Nowacki M, Nuñez I, Pain A, Piersanti A, Pucciarelli S, Pyrih J, Rest JS, Rius M, Robertson D, Ruaud A, Ruiz-Trillo I, Sigg MA, Silver PA, Slamovits CH, Jason Smith G, Sprecher BN, Stern R, Swart EC, Tsaousis AD, Tsypin L, Turkewitz A, Turnšek J, Valach M, Vergé V, von Dassow P, von der Haar T, Waller RF, Wang L, Wen X, Wheeler G, Woods A, Zhang H, Mock T, Worden AZ, Lukeš J (2020). Genetic tool development in marine protists: emerging model organisms for experimental cell biology. Nat Methods.

[66] Fukuda K, Cooney EC, Irwin NAT, Keeling PJ, Hirakawa Y (2020). High-efficiency transformation of the chlorarachniophyte *Amorphochlora amoebiformis* by electroporation. Algal Res.

[67] Coordinates and structure factors in Protein Data Bank. https://identifiers.org/pdb:7C5V Released 28 Apr 2021.

[68] Coordinates and structure factors in Protein Data Bank. https://identifiers.org/pdb:7C5W Released 28 Apr 2021.

[69] Coordinates and structure factors in Protein Data Bank. https://identifiers.org/pdb:7C5X Released 28 Apr 2021.

[70] Coordinates and structure factors in Protein Data Bank. https://identifiers.org/pdb:7C5Y Released 28 Apr 2021.

