# Supplementary material for: Characterization of a novel type of carbonic anhydrase that acts without metal cofactors
Source: BMC Biol. 2021 May 18;19:105. doi: 10.1186/s12915-021-01039-8 (PMC8132391; doi:10.1186/s12915-021-01039-8)
Supplement: Supplementary file 2 — Additional file 2: Figure S1. Maximum-likelihood phylogenetic tree of COG4337 proteins. The tree was contracted with 214 of COG4337 domain sequences extracted from 30 prokaryotic and 102 eukaryotic proteins. Sequences of multiple repeated domains are labelled by the ordinal number. Numbers at nodes indicate bootstrap supports (BS) that are shown only when they are higher than 50%. Black dots correspond to ≥95% BS. The scale bar represents the expected number of amino acid substitutions per site. Figure S2. Sequence alignment of COG4337 and COG4875 domains. The alignment includes COG4337 domains extracted from Bn86287 (JGI Bigna1: 86287), Bn50950 (JGI Bigna1: 50950), and all2909 (GenBank: BAB74608), and COG4875 domains of LCIP63 (JGI Thaps3: 9854) and 3H51 (GenBank: AAM40142). Numbers next to protein names represent the position of repeated domains. Asterisks show conserved amino acids, and the C-terminal motif “His-His-Ser-Ser” is highlighted by a yellow box. Figure S3. Esterase activity. Esterase activity was measured with 4-nitrophenyl acetate as substrate. Absorption at 348 nm was monitored for 5 min after the addition of each protein at the time point 60 sec. Values of esterase activity are summarized in the table (mean ± SD of three independent experiments). BSA, bovine serum albumin. Figure S4. SEC-MALS analysis of recombinant COG4337 proteins. (A, B) Light scattering (LS, red line), differential reflective index (dRI, blue line), and the molecular weight of the protein (black line) are plotted against the elution volume. Theoretical molar mass of the Bn82787 and all2909 monomer being 55.3 kDa and 19.3 kDa, respectively. Bn86287 and all2909 were estimated to exist as dimers and tetramers in solution, respectively. (C) Analysis with the PISA (Protein Interfaces, Surfaces and Assemblies) software estimated that a tetramer of all2909 was assembled by a head-to-head interaction of two dimeric units. Figure S5. Structural comparison of COG4337 domains. (A) Structur [file 12915_2021_1039_MOESM2_ESM.pdf]

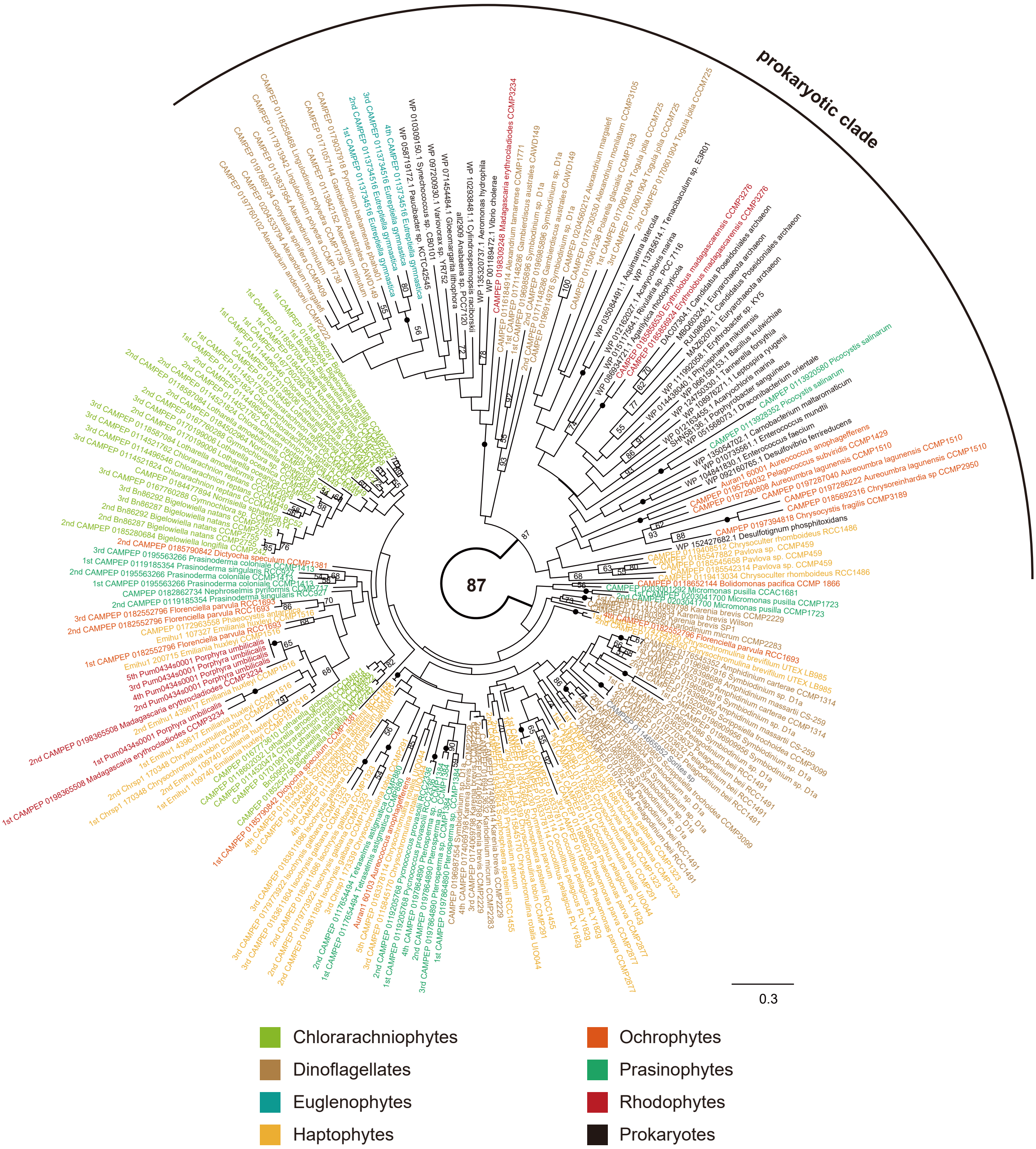

Fig S1. Maximum-likelihood phylogenetic tree of COG4337 proteins. The tree was contracted with 214 of COG4337 domain sequences extracted from 30 prokaryotic and 102 eukaryotic proteins. Sequences of multiple repeated domains are labelled by the ordinal number. Numbers at nodes indicate bootstrap supports (BS) that are shown only when they are higher than 50%. Black dots correspond to ≥95% BS. The scale bar represents the expected number of amino acid substitutions per site.

```
COG4337  Bn86287-1    ITEAEVLNAQSKWAEAIKTISRTYLNGGDYIKTAGDAA--AELYGYGKSKVLFKPTKA-A
COG4337  Bn86287-2    VTLKEVTECQEKWANAIQTISKTYLDGGDYIGEAGKQA--GILYGYGNTNVLFKPTKA-T
COG4337  Bn86287-3    ITEAEVLECQKNWANAIQTISKTYLDGGDYIGEAGKQA--GILYGYGNTNVLFKPTKA-T
COG4337  Bn50950      VTEADVLECQDKWATAIKTISKTYLEKGDFVGAAGAAA--GELYGYGHTNVLFKPTKA-A
COG4337  all2909      ITESEVLAAQKAWGEALVAISTTYDAKGKASAKALAEKVIDDAYGYQFGPVLFKPTLAIS
COG4875  LCIP63-1     LF--------TLWNSALATGDSR-----------------IVASRYTKNPVLL-PTVS--
COG4875  LCIP63-2     LF--------QLWNSALATEDPD-----------------AVAARYSNNAVLL-PTVS--
COG4875  LCIP63-3     LF--------NLWNDALATKDPI-----------------QVAKRYSKDGVLL-PTVS--
COG4875  LCIP63-4     LF--------SLWNNALATLDPK-----------------QVAARYAKKGVLL-PTVS--
COG4875  3H51         LF--------DTWNAALATGNPH-----------------KVADLYAPDGVLL-PTVS--
                                  *  *                             *    **  **

         Bn86287-1    EFPFRPTGEEAMSYFVGGNAVEKGY-KEDAGFAINGGKGWSNVVFNNHDID-INGNTAVA
         Bn86287-2    DHPFRPTGEQAMSYFVGGDVVDNGYVGEDAGFAINGGKGWSKVVFRNHQVD-LNGPVAIA
         Bn86287-3    DHPFRPTGEEAMSYFVGGDVVENGYVGEDAGFAINGGKGWKNVVFRNHQLD-FNGPVAIA
         Bn50950      EYPFRPTGGEAMSYFVGGSNVDGGY-EEDGGFAINGGNGWKEVVFTNHQID-LSGPVAIA
         all2909      PRTFRTTRAGALAYFVGD---DKAF-PEDKGFALS---SWRKVEIKNAAIF-ITGNTATT
         LCIP63-1     -DQARTDYDSVKDYFDA--------------FLLKKPQG----KIIEGKIN-IGDSWASD
         LCIP63-2     -DVPRNSYALIKDYFVG--------------FLKRKPQG----TILESNVT-VGHNWASD
         LCIP63-3     -DDVRTDFPGIVDYFTN--------------FLKLEPQG----EILGGKVT-IGTNWAQD
         LCIP63-4     -DKARTDFSSIEDYFVN--------------FLKLKPRG----TILESHVT-VGKNWCQD
         3H51         -NEVRASREQIENYFEM--------------FLTKKPKG----VINYRTVRLLDDDSAVD
                          *        **                *

         Bn86287-1    MGSYVFTCATTGTE---TKVEYTFGYKRNDDGKVRIFLHHSSV-PY
         Bn86287-2    MGDYVFTSAADGSE---TRVEYTFGYKRNDDGNVRIFVHHSSV-PY
         Bn86287-3    MGDYVFTSAADNSE---TRVEYTFGYKRNPDGKPRIFLHHSSV-PY
         Bn50950      MGSYVFTCATTGDE---AKVEYTFGYKRNDDGKVRIFLHHSSV-PY
         all2909      MGNVIIT-DKQGKA---TTVDKTWQFLKDDHGKLRIITHHSSL-PY
         LCIP63-1     CGIYEFTLGATGEK---VKARYSFNYVQ-ENGVWKIQHHHSSVMPE
         LCIP63-2     AGVYEFTMGDNGDK---VKGRYSFVYVF-EDGQWKISHHHSSVMPE
         LCIP63-3     AGIYEFTMGATGQK---VRGRYTYVYVY-EDGEWKIQNHHSSVMPE
         LCIP63-4     AGIYEFEMRATGKT---VKGRYSFIYVY-EDGEWKINHHHSSIMPE
         3H51         AGVYTFTLTDKNGKKSDVQARYTFVYEK-RDGKWLIINHHSSAMPE
                       *                             *   *  ****  *
```

Fig S2. Sequence alignment of COG4337 and COG4875 domains. The alignment includes COG4337 domains extracted from Bn86287 (JGI Bigna1: 86287), Bn50950 (JGI Bigna1: 50950), and all2909 (GenBank: BAB74608), and COG4875 domains of LCIP63 (JGI Thaps3: 9854) and 3H51 (GenBank: AAM40142). Numbers next to protein names represent the position of repeated domains. Asterisks show conserved amino acids, and the C-terminal motif "His-His-Ser-Ser" is highlighted by a yellow box.

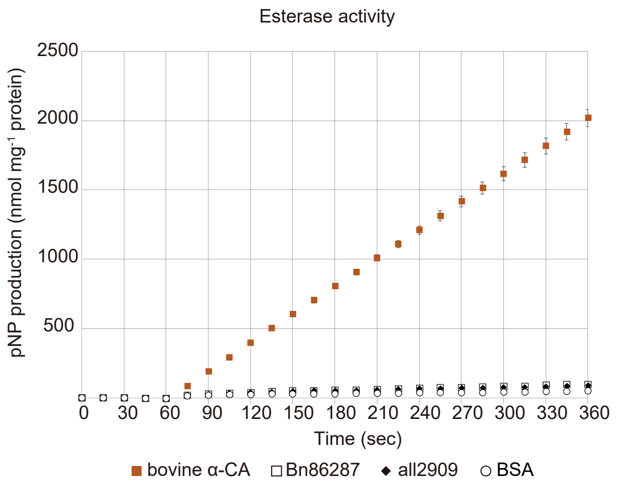

Protein	Esterase activity (nmol min^{-1} mg^{-1})
JGI86287	20.1 ± 0.5
all2909	18.4 ± 1.0
BSA	10.3 ± 0.9
Bovine α-CA	404.6 ± 11.7

Fig S3. Esterase activity. Esterase activity was measured with 4-nitrophenyl acetate as substrate. Absorption at 348 nm was monitored for 5 min after the addition of each protein at the time point 60 sec. Values of esterase activity are summarized in the table (mean ± SD of three independent experiments). BSA, bovine serum albumin.

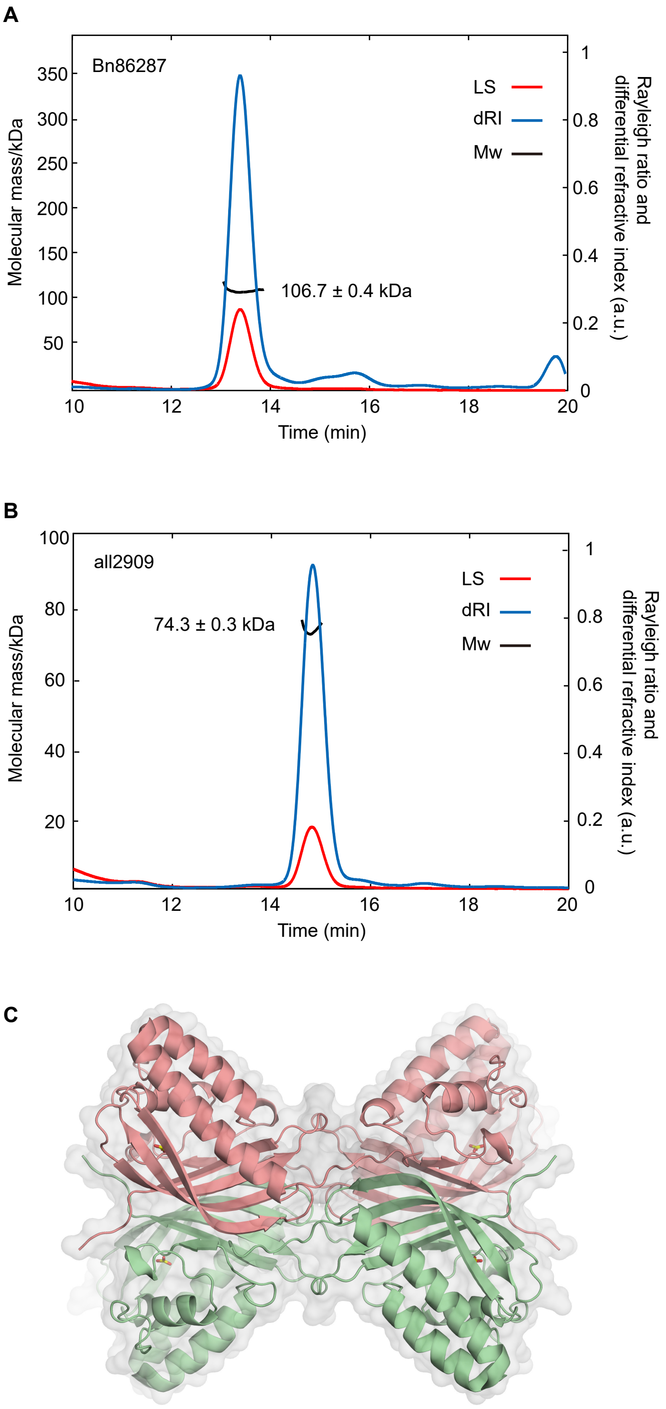

Fig S4. SEC-MALS analysis of recombinant COG4337 proteins. (A, **B)** Light scattering (LS, red line), differential reflective index (dRI, blue line), and the molecular weight of the protein (black line) are plotted against the elution volume. Theoretical molar mass of the Bn82787 and all2909 monomer being 55.3 kDa and 19.3 kDa, respectively. Bn86287 and all2909 were estimated to exist as dimers and tetramers in solution, respectively. **(C)** Analysis with the PISA (Protein Interfaces, Surfaces and Assemblies) software estimated that a tetramer of all2909 was assembled by a head-to-head interaction of two dimeric units.

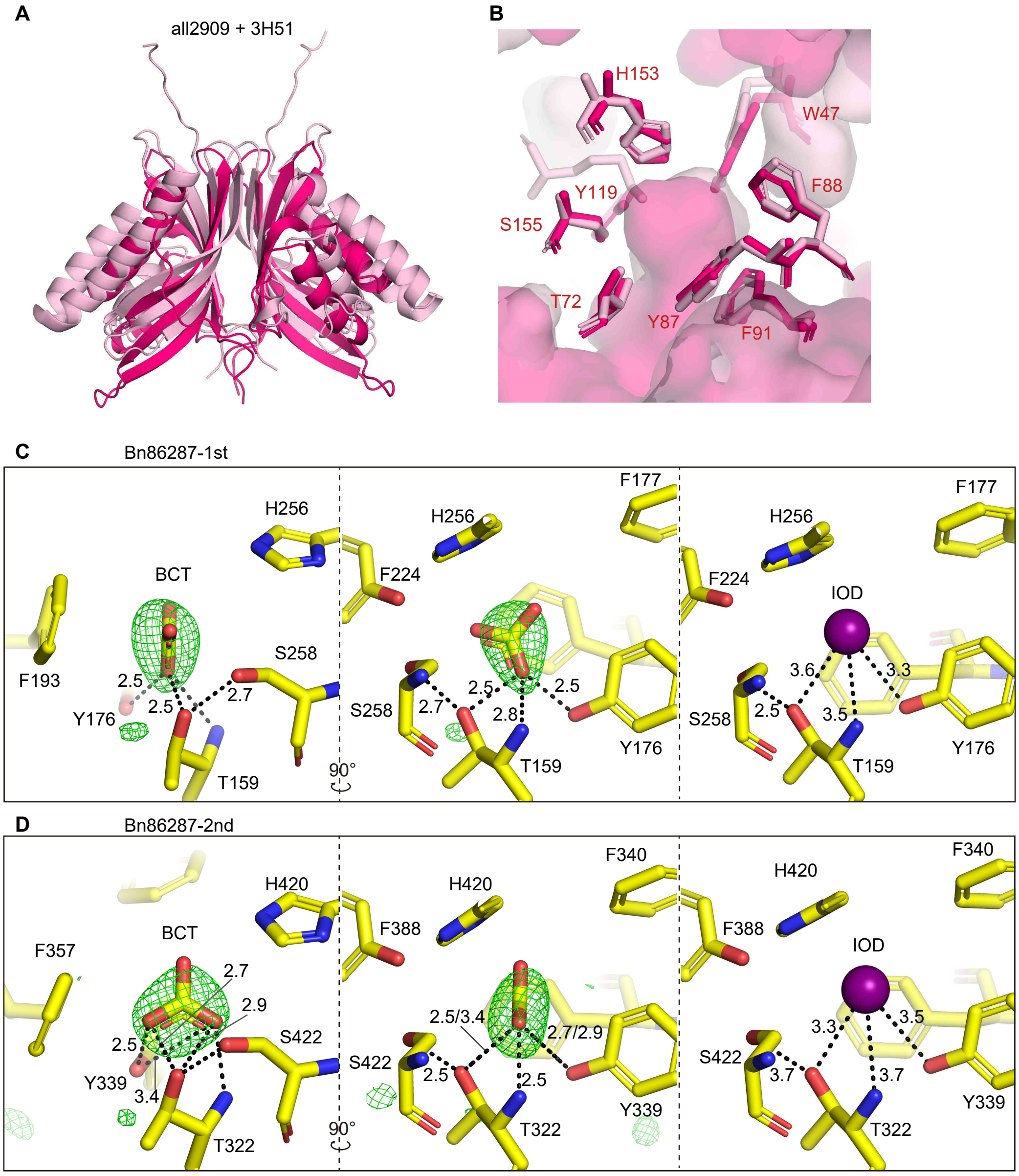

Fig S5. Structural comparison of COG4337 domains. (A) Structural alignment of the all2909 (dark pink) and an uncharacterized protein of *Xanthomonas campestris* (PDB ID: 3H51) (dark pink). **(B)** Cavities of all2909 and 3H51 are constructed by almost identical residues. **(C, D)** Active sites of the 1st and 2nd COG4337 domain in Bn86287. Simulated annealing Fo-Fc omit maps (green) for bicarbonate (BCT) are displayed at a contour level of 3.0 σ. Relevant distances between bicarbonate oxygen atoms/iodide (IOD) and neighbor residues are indicated by dashed lines with numbers. All images were prepared with PyMOL v. 2.3.3 (Schrödinger)

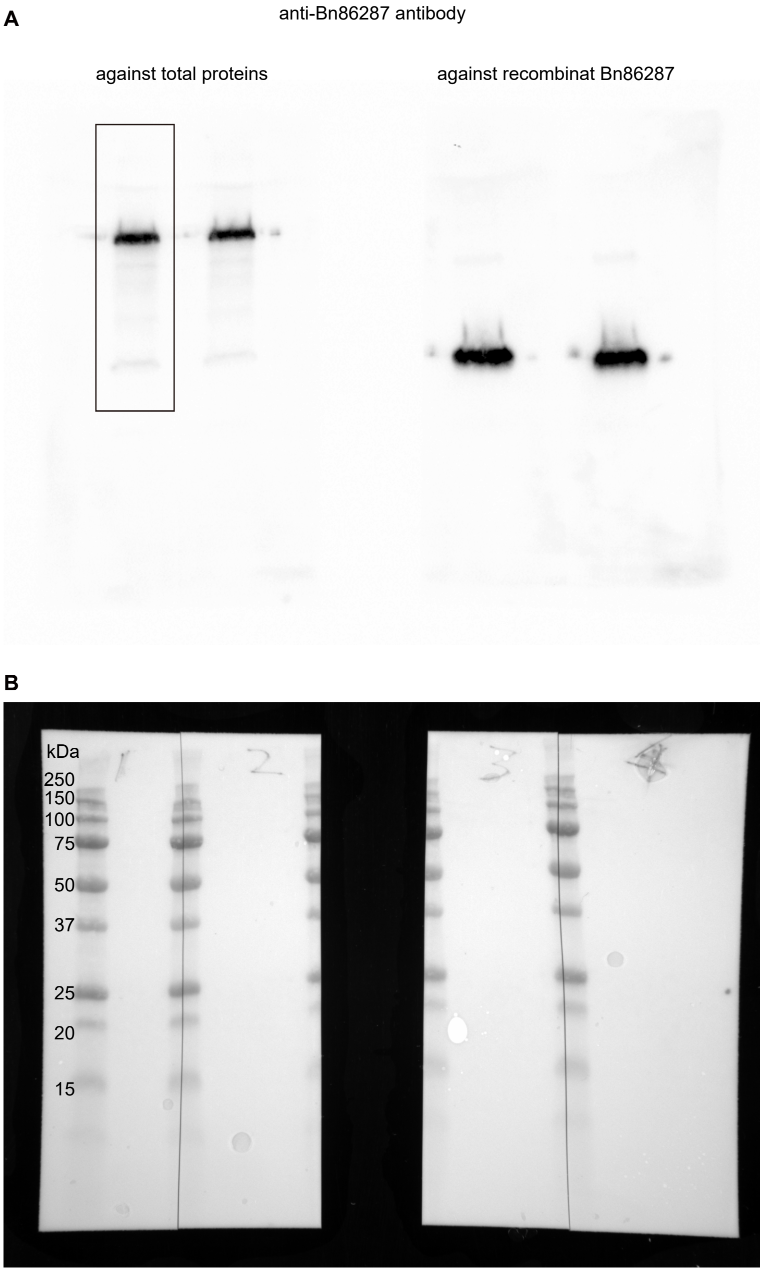

Fig S6. Uncropped immunoblotting images. (A) Western blots against total proteins and the recombinant Bn86287 protein. The square shows the cropped region for Fig 4A. **(B)** Brightfield image of the membrane.
