# Supplementary material for: Characterization of a novel type of carbonic anhydrase that acts without metal cofactors
Source: BMC Biol. 2021 May 18;19:105. doi: 10.1186/s12915-021-01039-8 (PMC8132391; doi:10.1186/s12915-021-01039-8)
Supplement: Supplementary file 3 — Additional file 3: Table S2. ICP-OES (inductively coupled plasma optical emission spectroscopy) analysis of six metals. Table S3. Primer sequences for plastid construction. [file 12915_2021_1039_MOESM3_ESM.pdf]

Table S2. ICP-OES (inductively coupled plasma optical emission spectroscopy) analysis of six metals.

	concentrations						
Sample	Protein (mg/ml)	Mg (ppm)	Ca (ppm)	Mn (ppm)	Co (ppm)	Zn (ppm)	Cd (ppm)
bovine CA	1.4	0.26±0.00	0.93±0.03	<MLOQ	<MLOQ	5.49±0.05	<MLOQ
Bn86287	1.2	0.06±0.01	0.77±0.03	<MLOQ	<MLOQ	<MLOQ	<MLOQ
all2909	1.1	<MLOQ	0.26±0.01	<MLOQ	<MLOQ	<MLOQ	<MLOQ
buffer	-	0.15±0.00	0.33±0.04	<MLOQ	<MLOQ	<MLOQ	<MLOQ

Metal concentrations were calculated as ppm in original protein solutions. The buffer contained 20 mM Tris-HCl (pH 8.0) and 100 mM NaCl. The method limits of quantification (MLOQ) were calculated to be 0.05 ppm for Mg, 0.2 ppm for Ca, 0.1 ppm for Mn, 0.2 ppm for Co, 0.45 ppm for Zn, and 0.15 ppm for Cd. The SD was calculated from three to five detections.

Table S3. Primer sequences for plastid construction

plasmid construct	forward primer (5' to 3')	reverse primer (5' to 3')
pET28-all2909-T106A	CAAGCCTGCACTCGCCATATCTC	GCGAGTGCAGGCTTGAAAAGAAC
pET28-all2909-Y124A	TTTGGCAGCCTTTGTGGGGGACGA	CACAAAGGCTGCCAAAGCCCCTGC
pET28-all2909-K180A	GGTAGACGCGACTTGGCAGTTCTTG	GCCAAGTCGCGTCTACCGTGGTGGC
pET28-all2909-H197A	TATTACGGCTCACTCCTCACTACC	GGAGTGAGCCGTAATAATACGCAG
pET28-all2909-S199A	GCATCACGCCTCACTACCTTATG	AGTGAGGCGTGATGCGTAATAAT
pET28-Bn86287-T486A	TCAAGCCTGCAAAGGCCACTGACC	GGCCTTTGCAGGCTTGAAGAGTAC
pET28-Bn86287-Y503A	GCTATGTCTGCCTTCGTAGGAGGCGA	CTACGAAGGCAGACATAGCTTCTTCG
pET28-Bn86287-Y552A	TGGGAGACGCCGTTTTCACCTCTGCT	GTGAAAACGGCGTCTCCCATAGCAAT
pET28-Bn86287-H584A	ATCTTCCTCGCCCACTCCTCGGTGCC	GAGGAGTGGGCGAGGAAGATGCGTGG
pET28-Bn86287-S586A	CTCCACCACGCCTCGGTGCCTTACA	GCACCGAGGCGTGGTGGAGGAAGAT
